# Correlation between the Microstructures of Bonding Interfaces and the Shear Strength of Cu-to-Cu Joints Using (111)-Oriented and Nanotwinned Cu

**DOI:** 10.3390/ma11122368

**Published:** 2018-11-25

**Authors:** Jing-Ye Juang, Chia-Ling Lu, Yu-Jin Li, K. N. Tu, Chih Chen

**Affiliations:** 1 Department of Materials Science and Engineering, National Chiao Tung University, Hsinchu 30010, Taiwan; david.mse03g@g2.nctu.edu.tw (J.-Y.J.); chialing.mse96g@g2.nctu.edu.tw (C.-L.L.); r777719982003@yahoo.com.tw (Y.-J.L.); kntu@ucla.edu (K.N.T.); 2 Department of Materials Science and Engineering, University of California at Los Angeles, Los Angeles, CA 90095, USA

**Keywords:** Cu-to-Cu direct bonding, nanotwinned Cu, surface diffusion, grain growth, shear strength

## Abstract

Highly (111)-oriented Cu pillar-bumps were bonded to highly (111)-oriented Cu films at temperatures ranging from 200 °C/100 °C to 350 °C/100 °C in N_2_ ambient conditions. The microstructures of the bonded interfaces affected the shear strength performance of the bonded Cu joints. The bonded interfaces at 300 °C/100 °C and 350 °C/100 °C had far fewer voids than interfaces bonded at 200 °C/100 °C and 250 °C/100 °C. In addition, grain growth took place across the bonding interfaces at temperatures above 300 °C/100 °C. The corresponding orientation map (OIM) showed the preferred orientation of large grown grains to be <100>. Shear tests revealed that the fracture mode was brittle for joints bonded at 200 °C/100 °C, but became ductile after bonded above 300 °C/100 °C. Based on the results, we found that voids and grain growth behavior play import roles in the shear strength performance of bonded Cu joints.

## 1. Introduction

Cu-to-Cu direct bonding has attracted attention in advanced packaging technology because it has the potential to replace solder microbump interconnections in high-end electronic devices [1,2,3,4,5]. As packaging and chip technology continue to scale down in size, solder microbumps will encounter processing challenges because their pitch cannot be reduced below 20 μm [6,7,8,9]. Thus, it is essential to adopt alternative bonding materials and techniques to overcome the scaling issue. At the moment, Cu-to-Cu direct bonding appears to be the most promising solution for ultra-fine pitch packaging, because it can be fabricated below 1 μm and it has excellent electrical and thermal conduction. In addition, it has excellent reliability performance due to its high toughness, high strength, and ductile mechanical properties [10,11,12].

Currently, most studies on the mechanical properties of microbump interconnections have focused on lead or lead-free solder materials. Suganuma et al. reported on the microstructure of ternary lead-free solder alloys and the growth kinetics of intermetallic compounds [13]. Hu et al. and Chen et al. focused on shear strength performance of solder bumps under thermal aging on SnPb/Cu and Sn-Ag-Cu/Cu joints, respectively. It was reported that the formation of intermetallic compounds degrades the shear strength after thermal aging [14,15]. Zhao et al. conducted shear test studies on different electroplated solder bump structures, such as Sn/Ag, Ag/Sn, Ag/Sn/Ag. The results revealed that Sn/Ag bumps show the highest strength value; rod-like and continuous Cu_6_Sn_5_ IMC enhances the shear strength of solder bumps [16]. However, much less attention is being devoted to obtain the correlation between the microstructure of bonding interfaces and the mechanical properties of bonded Cu-to-Cu joints.

In this study, Cu-to-Cu direct bonding experiments were performed in a N_2_-purged atmosphere with different bonding temperatures. The microstructure bonding interfaces were then analyzed. The novelty of this study is that we adopted highly (111)-oriented nanotwinned Cu (nt-Cu) and take advantages of its high surface diffusivity [17,18,19]. Therefore, the Cu direct bonding can be achieved at no-vacuum ambient conditions. In addition, (111) nt-Cu microbumps can be fabricated; they were bonded to nt-Cu films so that shear tests can be performed on the individual microbump. After the shear tests, fracture modes were examined. The correlation between the microstructures of bonding interfaces and the mechanical properties of bonded Cu joints were established.

## 2. Materials and Methods

The test vehicles consisted of top and bottom dies, which were designed to be amenable to the chip-to-chip or chip-to-wafer process. We prepared two types of Cu structures on the test dies. One was a Cu pillar bump array on the top die, and the other was a Cu thin film on the bottom die. Using this design, direct bonding could be achieved without chip alignment processes, and shear tests could also be performed. Highly (111)-nanotwinned Cu (nt-Cu) pillar bumps and films were fabricated to facilitate the Cu-to-Cu direct bonding. It has been reported that the (111) plane of Cu possesses a high surface diffusivity, so the unidirectionally-oriented (111)-nanotwinned Cu enables low temperature direct bonding via surface creep [17,18,19]. The electrodeposition procedures used for the (111) nt-Cu were reported in our previous publication [20].

**Figure 1:**
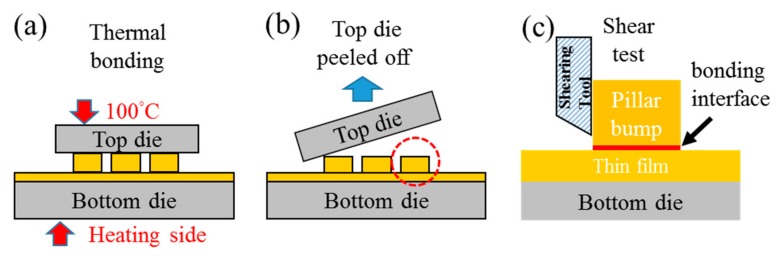
The schematic drawings show the experimental procedures. (**a**) Cu-to-Cu direct bonding, (**b**) top die peeled off, and (**c**) shear tests of Cu joint.

To perform shear tests, a small top die with Cu pillar bumps was bonded to a large bottom die with blanket-type Cu film. The size of the top die was 5 mm × 5 mm and the bottom die was 20 mm × 20 mm. The top die consisted of arrays of (111) nt-Cu pillar bumps, 30 μm in diameter. Both dies were planarized by chemical–mechanical polishing. Using an atomic force microscope (AFM, Veeco Dimension 3100, Bruker, Billerica, MA, USA) the measured root mean squared roughness (Rq) values were observed to be 5.12 nm and 1.34 nm for the nt-Cu pillar bump and nt-Cu thin film, respectively. Cleaning processes were performed to remove the organic contaminants and the oxide layer before bonding. The test dies were rinsed with deionized water, followed by a short immersion in a mixed solution of citric acid and deionized water (in the ratio: 133 g/100 mL) at 60 °C for around 30 s. They were rinsed again with deionized water and dried by N_2_ purging before bonding. The top and bottom die were bonded by thermal compression bonding at 40.6 MPa for 20 min at various temperature gradients as depicted in Figure 1a. The top die was kept at 100 °C, and the four different temperatures of 200 °C, 250 °C, 300 °C, and 350 °C were used on the bottom die during the bonding process.

Focused ion beam (FIB) was employed to observe the cross-section of the Cu-to-Cu bonded interface as well as grain growth in the bonded Cu joints. Subsequently, electron backscattered diffraction (EBSD) was performed to acquire Cu crystal orientation along the joint cross-section (JSM-7800F (JEOL, Tokyo, Japan) scanning electron microscope with Nordlys Max3 EBSD detector (Oxford Instruments, Abingdon, UK)). Aztec EBSD post-processing software was used to provide statistical orientation maps and crystallographic textures. Finally, to quantify the strength of the bonded Cu joints, shear tests were conducted. The tests were performed using a Nordson Dage-4000 shear tester (Nordson DAGE, Aylesbury, UK) with a BS250 testing module. To accurately measure the shear strength, the Si substrates on the top die were peeled off (Figure 1b), and shear tests were performed on the individual bonded Cu joints (Figure 1c). Tests were conducted at heights of 5 μm above the surface of Cu films, at a test speed of 100 μm/s. Twelve bonded Cu joints were shear tested at each temperature condition. Fracture modes of the shear tested Cu joints were observed and analyzed.

## 3. Results and Discussions

### 3.1. Grain Growth Evolution for Different Bonding Temperatures

**Figure 2:**
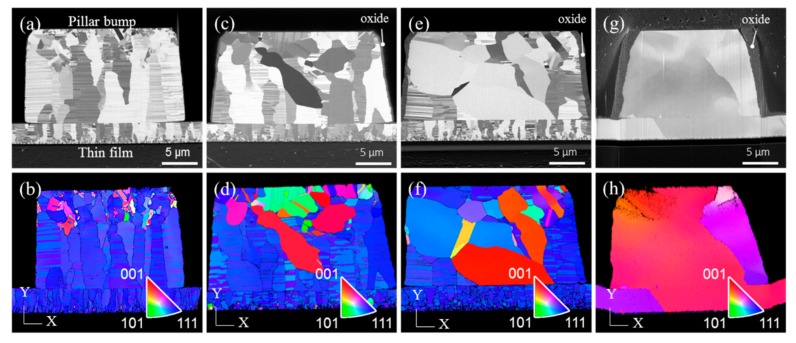
Cross-sectional focused ion beam (FIB) and electron backscatter diffraction (EBSD) images showing grain growth in the Cu joints bonded at various temperatures. (**a**) FIB image, 200 °C/100 °C; (**b**) EBSD image for the joint in (a); (**c**) FIB image, 250 °C/100 °C; (**d**) EBSD image for the joint in (c); (**e**) FIB image, 300 °C/100 °C; (**f**) EBSD image for the joint in (e); (**g**) FIB image, 350 °C/100 °C; and (**h**) EBSD image for the joint in (g).

Bonding between the Cu pillar bumps and the Cu films was achieved at a temperature gradient ranging from 200 °C/100 °C to 350 °C/100 °C in N_2_ ambient. Figure 2 presents the cross-sectional FIB and EBSD images for nt-Cu pillar bumps bonded to nt-Cu films. After bonding at a temperature condition of 200 °C/100 °C for 20 min, most of the nanotwinned columnar grains remained in the pillar bump and the Cu film, as shown in Figure 2a. Figure 2b shows the corresponding orientation map (OIM) and the pole figure, where the columnar grains were (111)-oriented. For the Cu joints bonded at 250 °C/100 °C, recrystallization and grain growth phenomena were observed, as shown in Figure 2c. Some of the nanotwinned grains disappeared and several grains, without nanotwins, grew in the Cu pillar bump near the Si substrate side. The new grains were <100>- and <110>-oriented, as presented in the cross-sectional OIM images in Figure 2d. Furthermore, grain growth was not found across the bonding interface, or in the Cu film. As the bonding temperature increased to 300 °C/100 °C, more columnar grains in the pillar bump were consumed by other grains, as depicted in Figure 1e,f. Extensive grain growth took place at 350 °C, as illustrated in Figure 2g,h. Large grains grew from the pillar bump to consume the (111) columnar grains in the Cu film. The orientations of the large grains were <100> and <211>, as shown in Figure 2h. It is noteworthy to point out that a thick oxide layer formed on the side wall of the Cu pillar bump after the bonding process at 350 °C, as labeled in Figure 2g.

### 3.2. Bonded Interfaces Characterization

**Figure 3:**
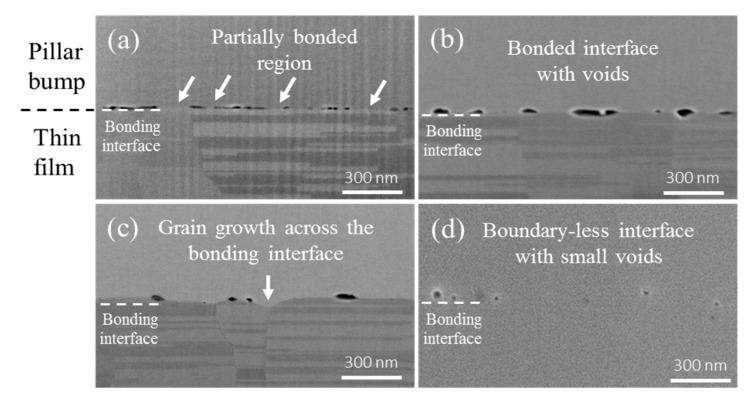
Cross-sectional secondary SEM images showing the microstructures in the bonding interface for the Cu joint bonded at (**a**) 200 °C/100 °C, (**b**) 250 °C/100 °C, (**c**) 300 °C/100 °C, and (**d**) 350 °C/100 °C.

Residual voids in the bonding interfaces were examined by Scanning electron microscope (SEM) images. Figure 3a–d present the microstructures in the bonding interfaces, bonded at 200 °C/100 °C, 250 °C/100 °C, 300 °C/100 °C, and 350 °C/100 °C, respectively. For the Cu joint bonded at 200 °C/100 °C, there were numerous voids along the interface. The partially bonded condition was observed (Figure 3a). As the temperature increased, more contact surface along the interface was bonded together. Thus, the number of voids decreased as the bonding temperature increased (Figure 3b). In addition, grain growth took place across the bonding interfaces at temperatures of 300 °C/100 °C (Figure 3c). The grown grains in the pillar bump side began merging with the lower nt-Cu columnar grains in the thin film side [21]. Finally, most nt-Cu columnar grains in the lower thin film side were consumed. Thus, a boundary-less interface with small voids was identified at the temperature condition of 350 °C/100 °C (Figure 3d). 

### 3.3. Shear Strength and Fracture Mode of the Bonded Cu Joints

**Figure 4:**
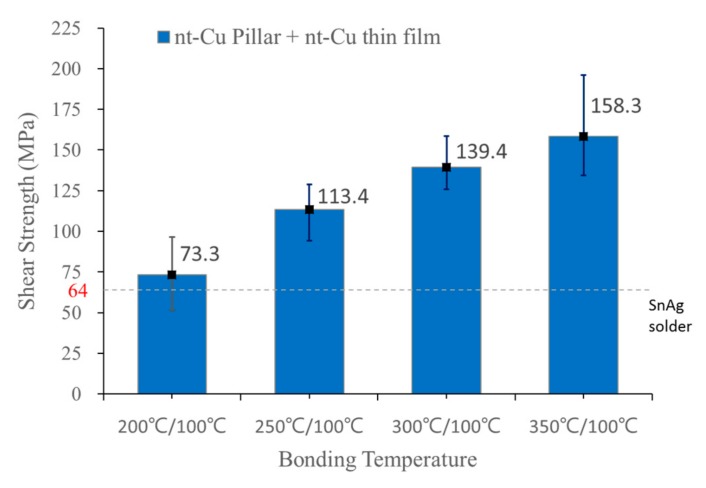
Plot of shear strength against bonding temperatures.

To quantify the strength of the Cu joints, shear tests were conducted. To measure the shear strength accurately, the Si substrates on the top die were first peeled off, then the individual bonded Cu joints underwent shear tests. Figure 4 shows measured shear strength against bonding temperature. The shear strength was only 73.3 MPa for Cu joints bonded at 200 °C/100 °C, however, strength increased as the bonding temperature increased. It reached 158.3 MPa for Cu joints bonded at 350 °C/100 °C. 

**Figure 5:**
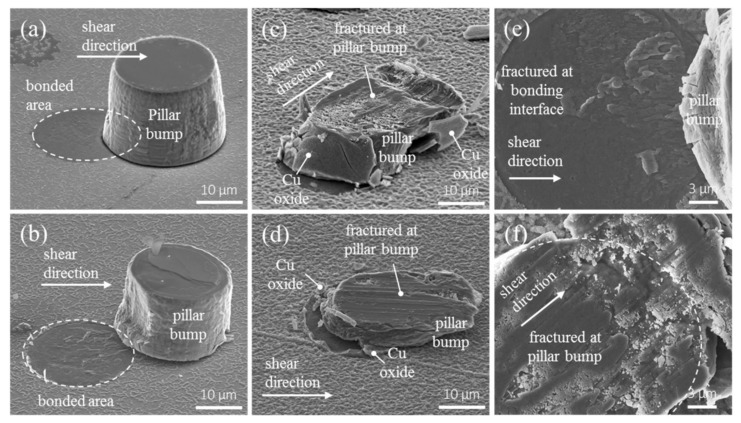
SEM images showing the fractures for the Cu joints bonded at (**a**) 200 °C/100 °C, (**b**) 250 °C/100 °C, (**c**) 300 °C/100 °C, and (**d**) 350 °C/100 °C. (**e**) Enlarged plan-view image for the fracture surface in (b). Brittle fracture occurred for the joints bonded at low temperature below 250 °C/100 °C, and (**f**) enlarged plan-view image for the fracture surface in (c). Ductile fracture took place above 300°C/100 °C.

The fracture modes were examined by SEM images. For Cu joints bonded at 200 °C/100 °C, the Cu pillar bump was shifted under the shear, and the fracture occurred at the bonding interface in a brittle manner, as shown in Figure 5a. In addition, there was almost no plastic deformation in the fracture interface. For the Cu joints bonded at 250 °C, a similar fracture mode was observed, as depicted in Figure 5b. Plastic deformation was also present in the pillar bumps and in the fracture interface. Conversely, as the bonding temperature increased to 300 °C/100 °C and 350 °C/100 °C, the fracture mode became ductile, and the fracture took place in the Cu pillar bumps, as seen in Figure 5c,d. The Cu oxides on the side walls of the Cu pillar bump delaminated from the bump, as indicated by some of the arrows in Figure 5c,d. Figure 5e shows the enlarged plan-view SEM image for a brittle failure surface for the Cu pillar bump bonded at 250 °C, whereas Figure 5f presents the enlarged top-view SEM image for the fracture surface in Figure 5c. Plastic deformation took place in the Cu pillar bump. Therefore, it is ductile fracture for the Cu direct bumps bonded at temperature above 300 °C. 

The voids and grain growth behavior in the bonding interface played critical roles in the fracture mode. As presented in Figure 2a,b, there were many voids in the bonding interface, and only a small percentage of the interfacial area had good bonding integrity. The average void size was 99 nm and 77 nm for Cu joints bonded at 200 °C/100 °C and 250 °C/100 °C, respectively. These Cu joints experienced brittle fracture. Yet, for the Cu joints bonded at 300 °C and above, much fewer and smaller voids existed in the interface and most of the interfacial area had excellent bonding. The average void size was 57 nm and 55 nm for Cu joints bonded at 300 °C/100 °C and 350 °C/100 °C, respectively. Therefore, ductile fracture occurred in the Cu pillar bump, instead of at the brittle interface.

## 4. Conclusions

In summary, the correlation between the microstructures of the bonding interface and the mechanical properties of the bonded Cu joints was established. With rapid surface diffusion on (111) surfaces, Cu-to-Cu direct bonding can be achieved under the temperature gradient, without vacuum ambient. Cu joints bonded at 200 °C/100 °C, and 250 °C/100°C show bonding interfaces with numerous voids and a brittle fracture mode at the bonding interface. Weak bonded joint conditions are observed. As the temperature increases to 300 °C/100 °C and then 350 °C/100 °C, the number of voids decreases, grain growth takes place across the bonding interfaces, and high shear strength values are obtained. At these temperatures the fracture mode occurs in a ductile manner. Based on the results, we found that voids and grain growth behavior play import roles on the shear strength performance of bonded Cu joints.
